# Dissolution, diffusion, and precipitation of Mg^2+^ in MgO-based cements, a review by the RILEM TC-311 MBC

**DOI:** 10.1617/s11527-026-03048-x

**Published:** 2026-03-23

**Authors:** Juan Pablo Gevaudan, Cristina Ruiz-Agudo, Hegoi Manzano, Hoang Nguyen, Barbara Lothenbach, Paivo Kinnunen, Ellina Bernard

**Affiliations:** 1 Department of Architectural Engineering, The Pennsylvania State University, University Park, PA 16802 USA; 2 Department of Materials Science and Engineering, The Pennsylvania State University, University Park, PA 16802 USA; 3 University of Konstanz, Constance, Germany; 4 Department of Physics, University of the Basque Country UPV/EHU, Barrio Sarriena s/n., 48940 Leioa, Spain; 5 University of Oulu, Oulu, Finland; 6 Swiss Federal Laboratories for Materials Science and Technology (Empa), Dübendorf, Switzerland

**Keywords:** MgO-based cements, MgO hydration, Brucite, Mg(OH)₂, Nucleation and growth, Diffusion, Magnesium silicate hydrate (M–S–H)

## Abstract

Magnesia-based cements (MBCs) are promising low-carbon alternatives to conventional portland cement due to their lower calcination temperature requirements and ability to utilize magnesia derived from carbon-free feedstocks. Understanding these systems requires deep knowledge of the entire reaction sequence: from the initial dissolution of magnesia (MgO) particles and the transport of magnesium ions through solution, to the precipitation of brucite [Mg(OH)₂], and ultimately of binding phases such as magnesium silicate hydrates (M–S–H) or magnesium carbonates. While these reaction steps resemble those in CaO-based systems, fundamental differences in thermodynamic and kinetic behaviors result in key differences in the dissolution and precipitation pathways of MBCs. To further explicate these differences, this review utilizes thermodynamic simulations revealing the effect of chemical additives (complexing ligands) on the solubility of Mg(OH)_2_, as the main intermediate phase in the precipitation of MBC binding phases. The inherent low solubility of Mg(OH)₂ is a key limitation that, upon further understanding and tailoring with chemical additives, can tailor the molecular pathways of Mg dissolution and transport, leading to controlled growth—paramount for optimizing strength development and durability in MgO-based cementitious materials.

**Supplementary Information:**

The online version contains supplementary material available at https://doi.org/10.1617/s11527-026-03048-x.

## Introduction

Across global priorities, decarbonizing the concrete sector is paramount, and the utilization of alternative cements has been proposed as a key strategy in these efforts. Amongst the toolkit of alternative cements, MgO-based cements (MBCs) are a special class that has gained research attention in the concrete sector, and unique applications, namely nuclear waste disposal, infrastructure repair, and specialized building products [1]. These applications benefit from advantageous material properties, including low-pH pore solution [2, 3] or high strength [4, 5]. While these properties change depending on the formulation of MBCs, the primary constituent of these cements, magnesia (MgO, periclase), plays a crucial role. In this context, MgO hydration is a complex phenomenon affected by the aqueous chemistry of the initial solution and leads to the precipitation of cementitious phases with varying solubilities.

When considering their global warming potential, carbon neutrality is often touted due to the minimal emissions associated with new processes for MgO extraction, such as electrochemical extraction of magnesia from seawater [6, 7], saline brine [8–10], or magnesium silicate minerals [11, 12]. These circumvent the reliance on calcination, which, when considering magnesium carbonates (MgCO_3_), emits higher CO_2_ than the production of portland cement due to the calcination of limestone [13]. In current commercial applications, MgO manufacturing involves the calcination of magnesium-containing raw minerals (e.g., magnesite, brucite) to produce a reactive product. When considering the dry production of MgO, the calcination temperature of magnesium carbonates can yield two distinct materials, namely light-burned (or caustic calcined magnesia) or dead-burned magnesia, also referred to as refractory magnesia. Light-burned magnesia, produced at calcination temperatures ranging from 700 to 1000 °C, is a highly reactive material characterized by a high surface area and micro-porosity, which reduces its density [14] when compared to dead-burned magnesia. The less reactive form of magnesia is produced at higher calcination temperatures (1500–2000 °C), resulting in a denser material with reduced surface area [15, 16], larger crystallite size, and a lower quantity of defects [16]. In addition to this manufacturing route, other processes have been reviewed by the authors elsewhere [1], where the purity of raw materials affects the produced crystal chemistry and its reactivity.

Differences in the raw materials and processing conditions result in varying physiochemical properties and, hence, influence the hydration reactions of MBCs. The high surface area, micro-porosity, and lower density of light-burned magnesia are well known to increase its hydration reaction rate and extent [14]. These physiochemical properties lead to a higher rate of dissolution of the light-burnt MgO compared to dead-burnt magnesia [16, 17]. Differences in physiochemistry of MgO produced from magnesite or brucite raw materials have also been reported. The MgO produced from brucite has been shown to have a higher surface area than that produced from magnesite at the same calcination temperature (900 °C). Changes in nanoporosity and surface hydroxylation [15, 16] are also important as they likely play a significant role in the extent and rate of MgO hydration and precipitation of cementitious phases, yet further detailed studies are needed to probe these differences.

The dissolution of magnesia can be described using the mineral dissolution theory, which addresses both atomic- and particle-scale processes [18–21]. From a chemical perspective, the overall hydration of MgO is often expressed by the generic *reaction *1. However, in practice, through-solution reactions unfold through multiple steps that span different length scales. At the atomic level, mineral dissolution theory describes how specific atoms (e.g., metal cations) detach from the lattice via chemical reactions [18, 19]. This includes the early hydroxylation steps (*reactions *2 and 3*,* where ≡ denotes a surface chemical bond), followed by detachment of Mg-dissolved species (*reaction *4), and finally the precipitation of Mg(OH)_2_ (*reaction *5). While these steps are widely accepted [22], various kinetic models propose intermediate phases and reactions to account for varying reaction rates at which MgO dissolves. On a larger, particle-scale level, mineral dissolution theory also considers how the surface morphology of MgO interacts with the surrounding solution [20, 21]. Here, factors such as surface roughness, defects, and pore solution chemistry can significantly influence the overall dissolution and hydration rate. Although the atomic- and particle-scale mechanisms are closely linked, the dominant rate-controlling step can vary depending on factors like MgO reactivity and processing conditions. In the following sections, we delve into recent findings on MgO hydroxylation, the diffusion pathways of dissolved Mg species, and the subsequent precipitation pathways of Mg(OH)_2_, highlighting how these processes collectively shape MgO hydration in MBCs.

1 $${\mathrm{MgO}} + {\mathrm{H}}_{{2}} {\mathrm{O}} \to {\mathrm{Mg}}\left( {{\mathrm{OH}}} \right)_{{2}}$$

2 $$\equiv {\mathrm{Mg}}{-}{\mathrm{O}} + {\mathrm{H}}_{{2}} {\mathrm{O}} { \leftrightharpoons } \equiv {\mathrm{MgOH}}^{ + } + {\mathrm{OH}}^{ - }_{{\left( {{\mathrm{aq}}} \right)}}$$

3 $$\equiv {\mathrm{MgOH}}^{ + } + {\mathrm{OH}}^{ - }_{{({\mathrm{aq}})}} { { \leftrightharpoons }} \equiv {\mathrm{MgOH}}^{ + } \cdot {\mathrm{OH}}^{ - }$$

4 $$\equiv {\mathrm{MgOH}}^{ + } \cdot {\mathrm{OH}}^{ - }{ { \leftrightharpoons }} {\text{ Mg}}^{{{2} + }}_{{\left( {{\mathrm{aq}}} \right)}} + {\mathrm{2OH}}^{ - }_{{({\mathrm{aq}})}}$$

5 $${\mathrm{Mg}}^{{{2} + }}_{{({\mathrm{aq}})}} + {\mathrm{2OH}}^{ - }_{{({\mathrm{aq}})}} { \leftrightharpoons } {\mathrm{Mg}}\left( {{\mathrm{OH}}} \right)_{{{2}\left( {\mathrm{s}} \right)}}$$

### ***Differences between MgO and CaO***

**Fig. 1:**
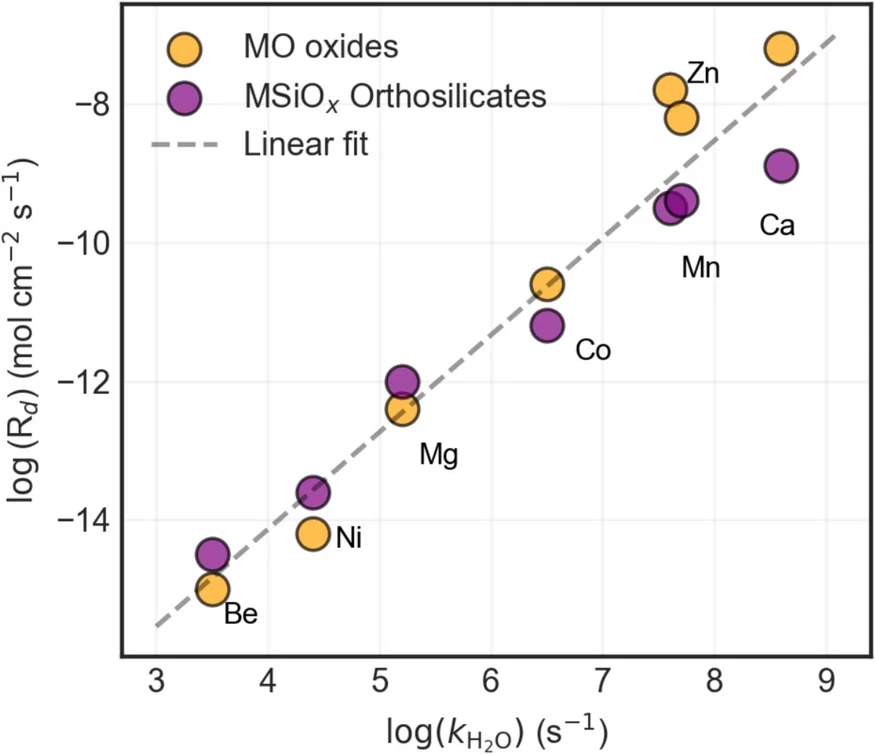
Correlation between metal-water exchange kinetics and acid dissolution rates (pH = 2.0) of divalent metal oxides (orange) and their orthosilicates (purple) at 25 °C. The intrinsic surface-normalized dissolution rate (R_d_) of well-ordered MgO is lower than that of CaO metal oxides by more than four orders of magnitude, while the rate constant for the first H_2_O exchange (k_H2O_) in the M(H_2_O)_n_.^2+^ aqueous complex is almost four orders of magnitude smaller. Adapted from [24]

Even though both MgO and CaO are isoelectronic materials with similar crystallographic structures, their hydration reactions show fundamental thermodynamic and kinetic differences. MgO and CaO crystals have similar atomic structures, as also seen in mineral pairs like magnesia and lime, brucite and portlandite, or sanderite and gypsum [23]. However, the dissolution rates of these oxides are vastly different, with highly-ordered fused MgO dissolving nearly two orders of magnitude slower than CaO [24], as shown in Fig. 1. Here, the dissolution rate is also observed to decrease linearly with the exchange rate of water into the hydration sphere of their aqueous ions (Mg^2+^, Ca^2+^). These findings align with recent ab initio molecular dynamics studies of a fully hydroxylated flat MgO(001) plane hydrating with multiple water layers. Within a limited simulation time (15 ps), MgO is shown to preserve its crystalline structure with a bilayer of hydroxyl groups at the Mg(001) plane and water interface [25]. When comparing the extent of the hydration and the surface evolution with other alkali earth oxides (i.e., Ca, Sr, Ba), this study confirms the retention of the MgO ordered crystalline structure, while for Ca, Sr, and Ba, the atomic arrangement at the surface is distorted upon hydration. In this context, further work is needed to understand how exposed MgO planes, including the influence of defects or uncoordinated sites, contribute to the hydration reactions of MBCs. While these contributions remain difficult to characterize due to differences in the stability of surfaces as physiochemistry changes, the lower reactivity of MgO can be attributed to a stronger Mg-O bond that increases the water dissociation energy barriers and, hence, decreases its water exchange rate.

While MgO and CaO undergo dissolution–precipitation reactions in nanometric water films [26], their different reactivities have prompted first-principles simulations to investigate the crystal chemistry origins of their contrasting behavior. Density functional theory (DFT) results indicate that for MgO(001), even the adsorption of a single water molecule is accompanied by a relatively high energy barrier. The water adsorption energy at the MgO surface has been reported to be ≈ − 52 kJ/mol (~ 0.54 eV) [27–29], followed by a dissociation into two hydroxyl groups with an energy barrier of ~ 1.1 eV (~ 106 kJ/mol). This energy barrier is surprisingly large, given the ionic nature of the Mg-O bond, and is comparable to the hydrolysis energy of Si–O–Si bonds [30]. In contrast, DFT calculations indicate that water dissociation is thermodynamically favorable at CaO surfaces, as reflected by an increased adsorption energy of − 92 kJ/mol [31]. For CaO, the facile dissociation of water rapidly produces hydroxylated, partially dissolved Ca(OH)_2_-like layers [32], which act as nucleation sites and accelerate subsequent growth processes [33]. These energetics highlight the fundamental thermodynamic and kinetic advantage that underpins higher-reactivity Ca-based cement systems.

Moreover, the Mg–O bond strength contributes to thermodynamic differences in both intermediate and final hydration products. For instance, Mg(OH)_2_ has a very low solubility of 0.0069 g/L (20ºC), which is approximately 230 times lower than the 1.6 g/L reported for Ca(OH)_2_ at neutral pH [34, 35]. Although solubility alone does not dictate hydration kinetics, it does hinder dissolution when Mg(OH)_2_ layers form on the MgO surfaces. 

### Poor diffusive flux

**Fig. 2:**
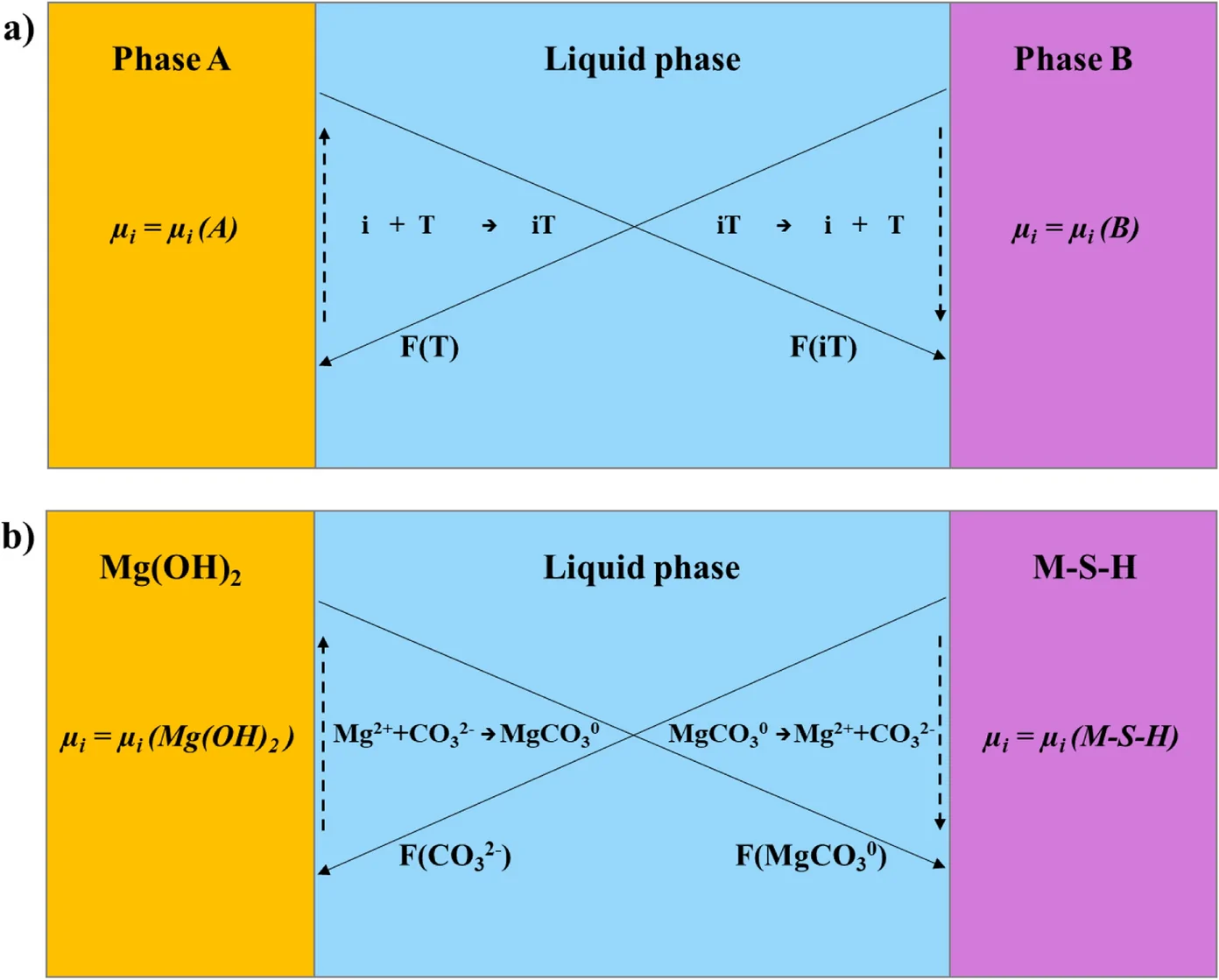
**a** Highly simplified presentation of how sustained diffusion can be attained using transport-enhancing chemical additives, where T corresponds to an empty ligand; i to a metal (oxide); and iT to aqueous ligand–metal complex. Given that diffusive flux (F) is proportional to the mean concentration (μ), the addition of T increases the total flux of the metal (i) if T and iT both possess higher average solubility than i and if the binding constant of the iT complex is such that the average value of the ratio [T]/[iT] is not too far from unity. (Figure adapted from presentation by prof. Ellis Gartner.) **b** the specific case of Mg(OH)_2_ dissolution in the presence of carbonates to form M–S–H

The rapid precipitation of poorly soluble Mg-phases [e.g., Mg(OH)_2_] is a key contributor to the poor diffusive flux of Mg-dissolved species (e.g., Mg^2+^). This occurs because diffusive flux depends directly on the concentration of dissolved species and their chemical potential gradient (Fig. 2). In MBCs, the heterogeneous nucleation and subsequent precipitation of low-solubility Mg-phases occurs at the dissolving surface and reduces the concentration of dissolved species. This, in turn, limits the establishment of aqueous chemical potential gradients and reduces the diffusive flux of dissolved species. Hence, the supply, transportation, and demand of dissolved species, as dictated by dissolution, flux, and precipitation processes, respectively, are critical to engineering the hydration mechanism of MBCs.

These diffusion fundamentals have motivated research to utilize chemical additives (complexing ligands) to increase the apparent solubility (i.e., the effective concentration) of Mg-dissolved species in MBCs, as will be discussed later. Figure 2 illustrates the capacity of complexing ligands (T) to increase the diffusive flux of dissolved species, as similarly explained to increase the solubility of iron species (Fe(III)) in clinker grains of portland cement by [36]. To maintain a high diffusive flux of the dissolved species, the aqueous chemical potential gradient must be maintained by (i) increasing the concentration of dissolved species near the dissolving surface, and (ii) enabling continuous precipitation away from this surface to preserve the gradient. As previously discussed, in MBCs, the solubility of precipitated phases is already extremely low, and, in high pH conditions, precipitates form at the dissolving surface, such as in the case of Mg(OH)_2_ [22, 37, 38]. This Mg(OH)_2_ passivating surface layer, therefore, yields a diffusion-limited hydration process, reducing the chemical potential gradient at the surface [39], and hence, points to the complex phenomena that limit the engineering of MBC hydration and cement-forming reactions. Therefore, to understand how this diffusive flux can be sustained using additives, the transportation of the dissolved species must be considered, as dictated by solubility.

### Scope of review: strategies for enhancing MgO hydration and solubility

While chemical additives, particularly organic complexing molecules containing hydroxyl, phosphate, and carboxyl groups [40–46], have demonstrated effectiveness in accelerating MgO hydration, the fundamental understanding of how these additives interact with MgO surfaces, influence magnesium ion mobility, and control Mg(OH)_2_ precipitation mechanisms remains partially lacking. The term precipitation is used as a general term to describe both nucleation and growth of a solid phase driven by supersaturation in the aqueous phase. This review examines the fundamental processes governing MBCs, focusing on Mg dissolution, transport, and the subsequent precipitation as Mg(OH)_2_, which ultimately leads to the precipitation of binding phases, such as magnesium silicate hydrate ( M–S–H). We explore the key steps underpinning MgO hydration, with attention to how chemical additives can help overcome the inherent limitations in each stage of the precipitation process. Our aim is to synthesize and analyze the existing literature on the transport and mobility of Mg-species in MBCs, integrating computational simulations and experimental investigations to propose strategies for enhancing the transport properties of Mg in these binders (Fig. 2) via the selection of the appropriate additives. Furthermore, we review the current understanding of both homogeneous and heterogeneous nucleation mechanisms of Mg(OH)₂ and M–S–H, delving into the key factors that influence their precipitation processes. We assess how various chemical additives impact the precipitation pathways, potentially modifying the materials' structural characteristics and long-term stability.

## MgO hydration

### Hydroxylation of the MgO surface

The initial step in converting MgO into Mg(OH)_2_ involves water dissociation and its adsorption onto the MgO surface [47]. During this surface hydroxylation process, often described by *reactions *2 and 3, water molecules split into hydroxyls that chemically bond to magnesium sites in a phenomenon referred to as *dissociative adsorption* [48]. Molecular dynamic studies [49, 50] have challenged the fully hydroxylated surface model at ambient temperatures, instead suggesting a more energetically favorable monolayer with hydroxyls located in bridging sites between Mg^2+^ ions. 

**Fig. 3:**
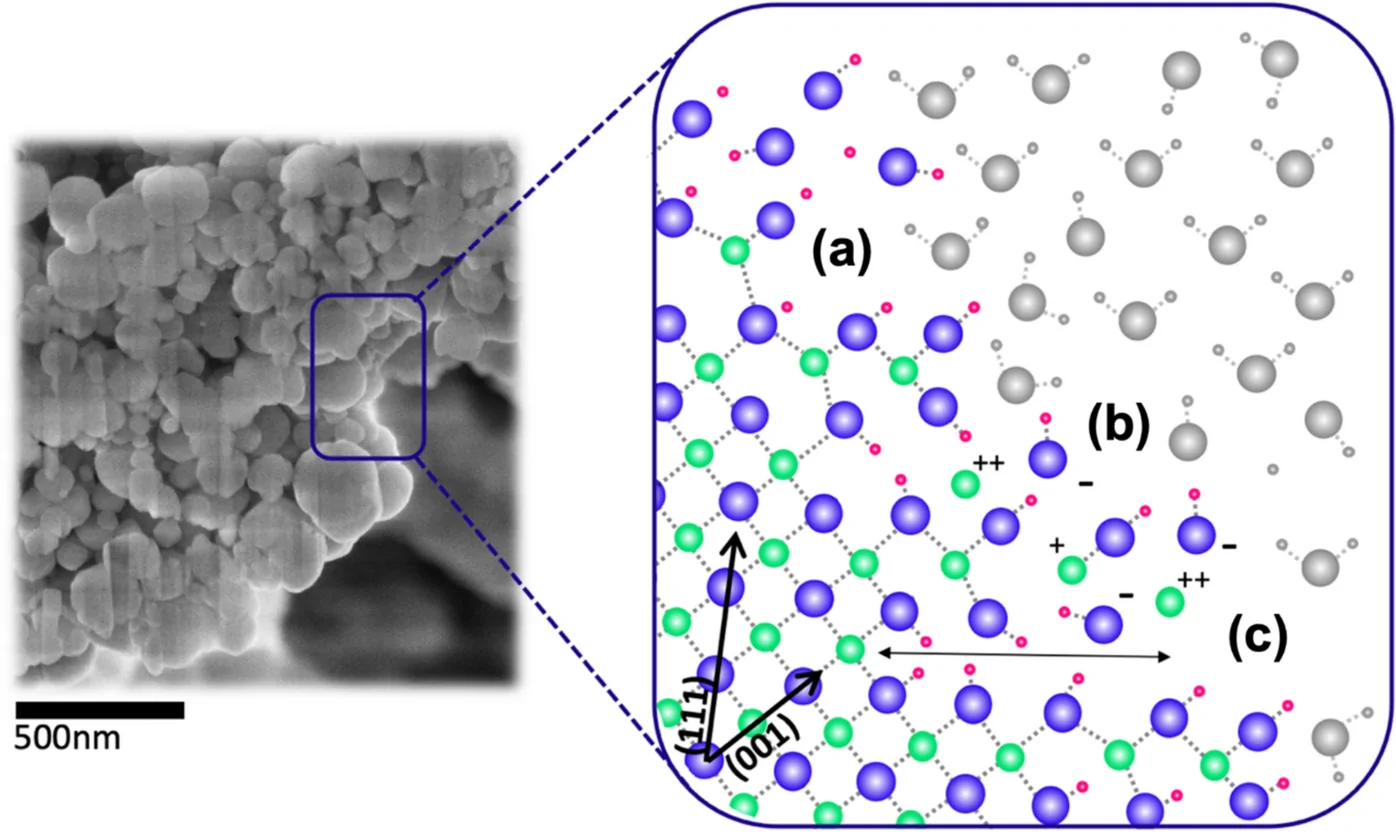
Electron microscopy image of nano-crystalline light-burned MgO (left). Atomistic sketch of the hydration mechanism of MgO (right), where the **a** dissociation of water at steps, kinks, and corners of the MgO(100) terrace leads to the surface hydroxylation. Proton-cation exchange enlarges these defects, enabling the **b** ion detachment and transport of solvated species (MgOH^+^, Mg^2+^) along concentration gradients. The accumulation of OH^−^, Mg^2+^, and MgOH^+^ species results in the **c** supersaturation with respect to Mg(OH)_2_. Heterogeneous nucleation of Mg(OH)_2_ is favored on the high-energy MgO(111) plane, where its rapid hydroxylation reconstructs the surface into a low-energy state and directs platelet growth normal to the Mg(OH)_2_(0001) plane. Schematic reaction based on references [49, 50], where magnesium (green), oxygen (blue), and hydrogen (red) atoms of the MgO structure are shown. Significant simplification of water films and electrolyte atomic arrangements is demonstrated here, with molecules represented in gray to facilitate discussion on MgO hydration

At the MgO-water interface, water dissociation plays a key role in governing the Mg^2+^ release since the degree of Mg^2+^ dissolution depends on how many water molecules adsorb and dissociate on the MgO surface [51]. Numerous studies show that water dissociation at MgO surface is only partial [52–57], meaning some water molecules donate protons to the surface, leaving OH^−^ ions near the interface. This process creates an electrical double layer, where the MgO surface carries a net positive charge balanced by nearby OH^−^ ions [58]. Concurrently, hydroxyl groups form on the surface, and their hydrogen-bonding arrangements continue to be a subject of active research to understand hydrophilic interactions at the MgO-water boundary [59]. Recent work [60] suggests that hydroxyls can engage in both direct (non-hydrogen) and hydrogen bonding, including interactions with OH^−^ ions formed from water dissociation (Fig. 3a). Moreover, the thermodynamic favorability of these hydroxylation processes can depend on particle topology; for instance, computational simulations indicate that steps along the MgO(001) plane are more readily hydroxylated than flat regions [61]. Adjusting the pH can further catalyze these hydroxylation reactions. However, such hydroxylated surfaces do not always represent the most stable structural configuration.

### Dissolution and diffusion of Mg-species

Recent studies have experimentally verified that MgO surfaces stabilize solvated Mg^2+^ ions [62], supporting previous computational findings [51]. As water adsorbs on the MgO(001) plane, the growing layer of hydroxyl groups renders the surface increasingly unstable, prompting the release of dissolved species (*reaction *4) [26]. Soft X-ray absorption spectroscopy experiments [62] confirmed that fully solvated Mg^2+^ ions, namely [Mg(H_2_O)_6_]^2+^, form at the MgO(001) interface during hydration and are energetically more favorable than a fully hydroxylated surface. This solvated Mg^2+^ formation represents the second stage in MgO dissolution (Fig. 3b). According to [51], when an Mg^2+^ ion leaves the surface, the charge imbalance is compensated by two newly formed hydroxyl groups, thus increasing local hydroxylation. The process is known as metal-proton exchange mechanism [63], and has been described for mineral oxides, and in the context of cement, for clinker phases [64, 65].

**Fig. 4:**
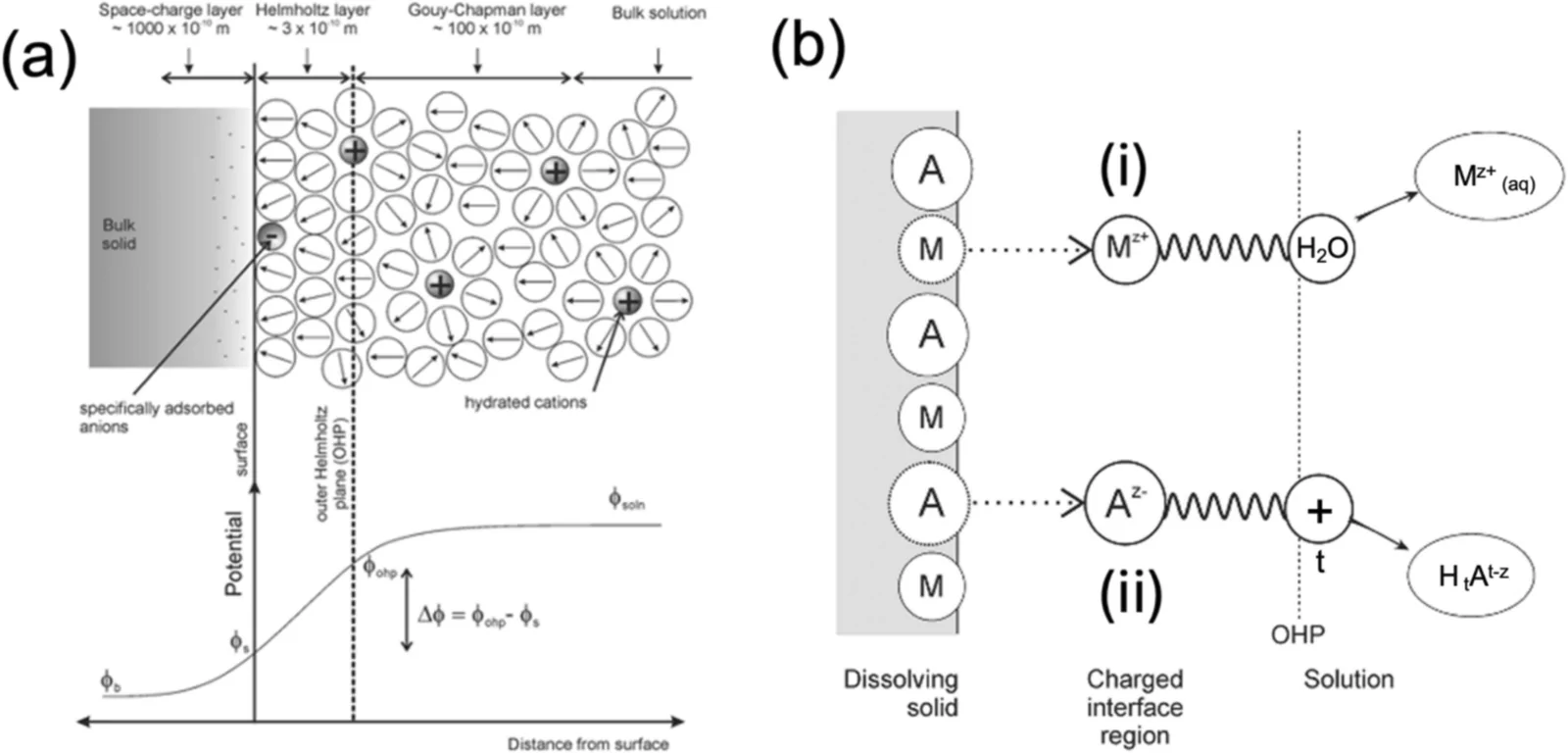
The structure of the mineral charge interface. **a** According to the surface vacancy model, the change in potential difference over the Helmholtz layer influences the kinetics of the reaction. Here, the symbol φ refers to the electrical potential, and the subscripts b, s, ohp, and soln refer to bulk solid, surface, outer Helmholtz plane, and bulk solution, respectively. **b** Dissolution mechanism in aqueous solution: (i) the bonds of magnesium (M) species with the surface break forming Mg^2+^_(aq)_ (M^z+^) ions; (ii) independently the bonds of oxygen (A) species with the surface break and O^2−^ reacts with H^+^ ions at the outer Helmholtz plane, resulting in the formation of OH^−^ or H_2_O (H_t_A.^t−z^). Reproduced with permission from [68]

In the surface-vacancy model [66], formation of solvated ions (*reaction *4 intermediates) are governed by the changing potential across the inner and outer Helmholtz layers (Fig. 4). Although fundamental in determining how efficiently Mg^2+^ species detach from the MgO/Mg(OH)_2_ surface, this mechanism remains understudied in the context of MBCs, with only a few studies probing the surface charge of MgO as it hydrates [67]. A better understanding of how surface charge affects dissolution rates could be critical for optimizing MgO dissolution rates, a key factor in formulating high-performance MBCs.

Additionally, magnesium on the MgO surface reacts at high pH with hydroxide ions to produce MgOH^+^ (*reaction *6). Simultaneously, oxygen at the surface combines with water to form hydroxide ions (*reaction *7). Accounting for these partial reactions helps clarify how MgO bonds break under hydration and influence the interfacial potential. For MBCs, understanding how these dissolved Mg species diffuse, leading to conditions favorable for the nucleation of Mg(OH)_2_ (*reaction* 8) is crucial for optimizing hydration rates.

MgO hydration and dissolution at alkaline conditions (high pH) based on the surface-vacancy model, where surface-bound atoms are denoted accordingly (≡) and accumulated charges on the surface are denoted as (≡^+^, ≡^−^).

6 $$\equiv {\mathrm{Mg}} + {\mathrm{OH}}^{ - } { { \leftrightharpoons }} \equiv^{{{2} - }} + {\mathrm{MgOH}}^{ + }$$

7 $$\equiv {\text{O }} + {\text{ H}}_{{2}} {\mathrm{O}}_{{({\mathrm{ads}})}} { { \leftrightharpoons }} \equiv^{{{2} + }} + {\text{ 2OH}}^{ - }$$

8 $${\mathrm{Mg}}^{{{2} + }}_{{({\mathrm{aq}})}} + {\mathrm{2OH}}^{ - }_{{({\mathrm{aq}})}} { \leftrightharpoons }{\mathrm{Mg}}\left( {{\mathrm{OH}}} \right)_{{{2}\left( {\mathrm{s}} \right)}}$$

Several approaches have been developed to enhance the dissolution of MgO, ranging from mechanical methods to chemical additives. Although not the most practical, one effective method is ultrasonic-assisted hydration. This method has been hypothesized to increase the rate of MgO hydration, through the physical effect of ultrasound [69, 70], where the cavitation energy possesses sufficient energy to break Mg–O bonds [71]. Once dissolved, the Mg species then diffuse along chemical potential gradients; however, their exact diffusion and speciation under real MBC conditions are still not fully understood and merit further investigation.

### The known Mg-complexes to increase the concentration of dissolved Mg

Another method to enhance the dissolution and diffusion of Mg^2+^ is the use of chemical additives to influence the kinetics, morphology, and crystallinity of the resulting Mg(OH)_2_ hydration product. Extensive literature demonstrates that magnesium-complexing ligands significantly enhance the degree of MgO hydration, compared to hydration in water alone. A wide range of chemical additives, including ammonium chloride (NH₄Cl), magnesium chloride (MgCl₂), magnesium nitrate (Mg(NO₃)₂), nitric acid (HNO₃), hydrochloric acid (HCl), and magnesium acetate (Mg(CH₃COO)₂), have been shown to increase hydration rates compared to distilled water e.g. [40–46]. Under acidic conditions, MgO and brucite dissolution proceeds primarily via proton-assisted surface reactions rather than diffusion-limited precipitation control. In this case, acidic conditions shift the chemical equilibrium towards the product side (Eqs. 2–4), enhancing the dissolution of MgO. While most of these studies have primarily focused on quantifying the degree of hydration, often without providing deeper insights into the underlying mechanisms, the observed enhancement has generally been attributed to the increased Mg concentration in solution, facilitated by complexation with dissolved ions and species as well as pH change.

We have gathered the Mg-complexes known in literature in Table 1 together with their stability constants (log *β*^*0*^_*m,l*_); a low stability constant indicates a weak complexation, while a high number indicates a strong complexation. The table indicates that, in particular, phosphate, citrate, and oxalate have the tendency to form strong aqueous complexes, while complexation of Mg^2+^ with monovalent anions such as Cl^−^, OH^−^ or acetate is rather weak, as discussed in more detail in Sect. 3.2.

**Table 1:** Complex formation constants for Mg^2+^ species collected from relevant literature

Species	Reaction	log *β*^*0*^_*m,l*_	References
MgOH^+^	Mg^2+^ + OH^−^ ominus MgOH^+^	2.56	[72]
MgCl^+^	Mg^2+^ + Cl^−^ ominus MgCl^+^	0.14	[73]
MgHSiO_3_^+^	Mg^2+^ + SiO(OH)_3_^−^ ominus MgSiO(OH)_3_^+^	1.50	[72]
MgSiO_3_^0^	Mg^2+^ + SiO_2_(OH)_2_^2^ ominus MgSiO_2_(OH)_2_^0^	5.70	[72]
MgSO_4_^0^	Mg^2+^ + SO_4_^2^ ominus MgSO_4_^0^	2.37	[72]
MgH_2_PO_4_^+^	Mg^2+^ + 2H^+^ + PO_4_^3−^ ominus MgH_2_PO_4_^+^	20.77	[74]
MgHPO_4_^0^	Mg^2+^ + H^+^ + PO_4_^3−^ ominus MgHPO_4_^0^	14.80	[74]
MgPO_4_^−^	Mg^2+^ + PO_4_^3−^ ominus MgPO_4_^−^	4.92	[74]
MgH_2_P_2_O_7_^0^	Mg^2+^ + 4H^+^ + 2PO_4_^3−^ ominus MgH_2_P_2_O_7_^0^ + H_2_O	40.62	[74]
MgHP_2_O_7_^−^	Mg^2+^ + 3H^+^ + 2PO_4_^3−^ ominus MgHP_2_O_7_^−^ + H_2_O	34.82	[74]
MgP_2_O_7_^2−^	Mg^2+^ + 2H^+^ + 2PO_4_^3−^ ominus MgP_2_O_7_^2−^ + H_2_O	28.51	[74]
MgHP_2_O_8_^3−^	Mg^2+^ + H^+^ + 2PO_4_^3−^ ominus MgHP_2_O_8_^3−^	16.61	[74]
MgAcetate^+^	Mg^2+^ + H_3_C_2_O_2_^−^ ominus MgH_3_C_2_O_2_^+^	1.28	[75]
Mg(Acetate)_2_^0^	Mg^2+^ + 2H_3_C_2_O_2_^−^ ominus Mg(H_3_C_2_O_2_)_2_^0^	2.04	[75]
MgHCO_3_^+^	Mg^2+^ + HCO_3_^−^ ominus MgHCO_3_^+^	1.07	[72]
MgCO_3_^0^	Mg^2+^ + CO_3_^2−^ ominus MgCO_3_^0^	2.98	[72]
MgOxalate^0^	Mg^2+^ + C_2_O_4_^2−^ ominus MgC_2_O_4_^0^	3.56	[76]
Mg(Oxalate)_2_^–2^	Mg^2+^ + 2C_2_O_4_^2−^ ominus Mg(C_2_O_4_)_2_^2−^	5.17	[76]
MgCitrate^−^	Mg^2+^ + C_6_H_3_O_2_^3−^ominus MgC_6_H_3_O_2_^+^	4.81	[76]
MgHCitrate^0^	Mg^2+^ + H^+^ + C_6_H_3_O_2_^3−^ ominus MgHC_6_H_3_O_2_^0^	2.60	[76]
MgH_2_Citrate^+^	Mg^2+^ + 2H^+^ + C_6_H_3_O_2_^3−^ ominus MgH_2_C_6_H_3_O_2_^+^	1.31	[76]

## Precipitation pathways of Mg(OH)_2_

The precipitation of brucite (Mg(OH)_2_) is a critical intermediate stage in MgO-based cement hydration. The characteristics of brucite, including its crystallinity, morphology, and whether it precipitates at the MgO surface or in the bulk solution, directly impact the binding phases that rely on Mg sourced from both MgO dissolution and brucite dissolution itself. Understanding its molecular-level precipitation is therefore essential for optimizing magnesium-based cements.

### MgO passivation problem

In the absence of chemical additives heterogeneous nucleation of Mg(OH)_2_ and at high pH levels (> ~ 12.0), at the MgO particle surface appears to be the predominant mechanism, with the characteristics of the resulting interfacial layer critically influencing MgO dissolution. As MgO hydrates and dissolves, the diffusion of solvated Mg ion species leads to their eventual adsorption onto Mg-OH nucleation sites at the surface, see Fig. 3c [49, 50]. This process represents the growth of surface Mg(OH)_2_ nanosheet nuclei [26, 77], which significantly slows or prevents the continued hydration of MgO to Mg(OH)_2_. Hence, the precipitation of this Mg(OH)_2_ passivation layer [69, 78–80] often results in a high content of residual MgO, commonly observed in MBCs, such as Mg-carbonate binders, even after 28 days [81, 82]. The residual MgO content in MBCs poses a key durability risk, as late-age hydration of MgO particle cores into Mg(OH)_2_ phases with a higher volume can induce expansion and crack formation [83].

**Fig. 5:**
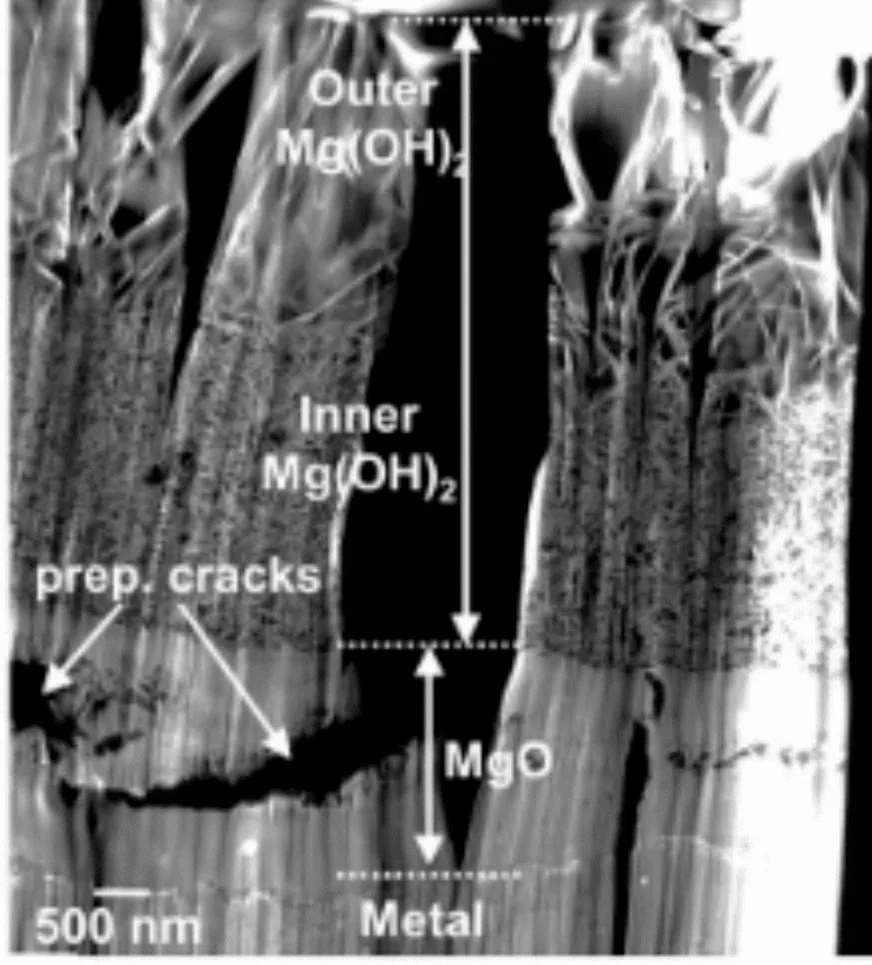
MgO–Mg(OH)_2_ (metal-film) interface imaged using multiple high-angle annular dark-field (HAADF) scanning transmission electron microscopy (STEM). The alloy metal Mg–Al–Zn (96, 3, 1 wt% respectively) here is exposed to D_2_O with 5 wt% NaCl for 24 h, and two distinct Mg(OH)_2_ layers are observed to form at the interface. Reproduced with permission from [84]

In the context of magnesium metal degradation in the presence of NaCl solutions, two types of Mg(OH)_2_ precipitates are observed at the surface of a Mg metal alloy, as shown in Fig. 5. Here, the outer and inner Mg(OH)_2_ layers differ in their porosity and morphology, likely suggesting distinct precipitation mechanisms or rates. While the metallic bonds and aqueous chemistry differ significantly from cement systems [84], computational [49, 50, 85] and experimental [77, 86] studies have also proposed the bidirectional growth of Mg(OH)_2_, resulting in the epitaxial relation between MgO(111) and Mg(OH)_2_(0001) crystallographic planes. The overall morphology of the MgO appears preserved, and a sharp interface between the MgO and the Mg(OH)_2_ can be observed, indicating that the two Mg(OH)₂ layers likely result from pseudomorphic replacement, where dissolution and precipitation processes are coupled both spatially and temporally [87]. The observed reduction in porosity when moving toward the interface between Mg(OH)_2_ and MgO represents a well-documented phenomenon in mineral replacement reactions, attributed to diffusion-limited transport of reactants through evolving pore networks, and consequent localized supersaturation gradients that influence precipitation kinetics [87]. Further investigations are needed to demonstrate the presence of inner and outer Mg(OH)_2_ layers during and the characteristics of their porosity in MBCs, as these layers may further prevent the complete hydration of remnant MgO cores. To enable complete dissolution of the MgO surface, thereby enhancing MBC hydration/carbonation and addressing the durability challenge, additional research is needed to control Mg(OH)_2_ growth and alter precipitate properties (e.g., morphology and porosity).

### The importance of understanding brucite precipitation

**Fig. 6:**
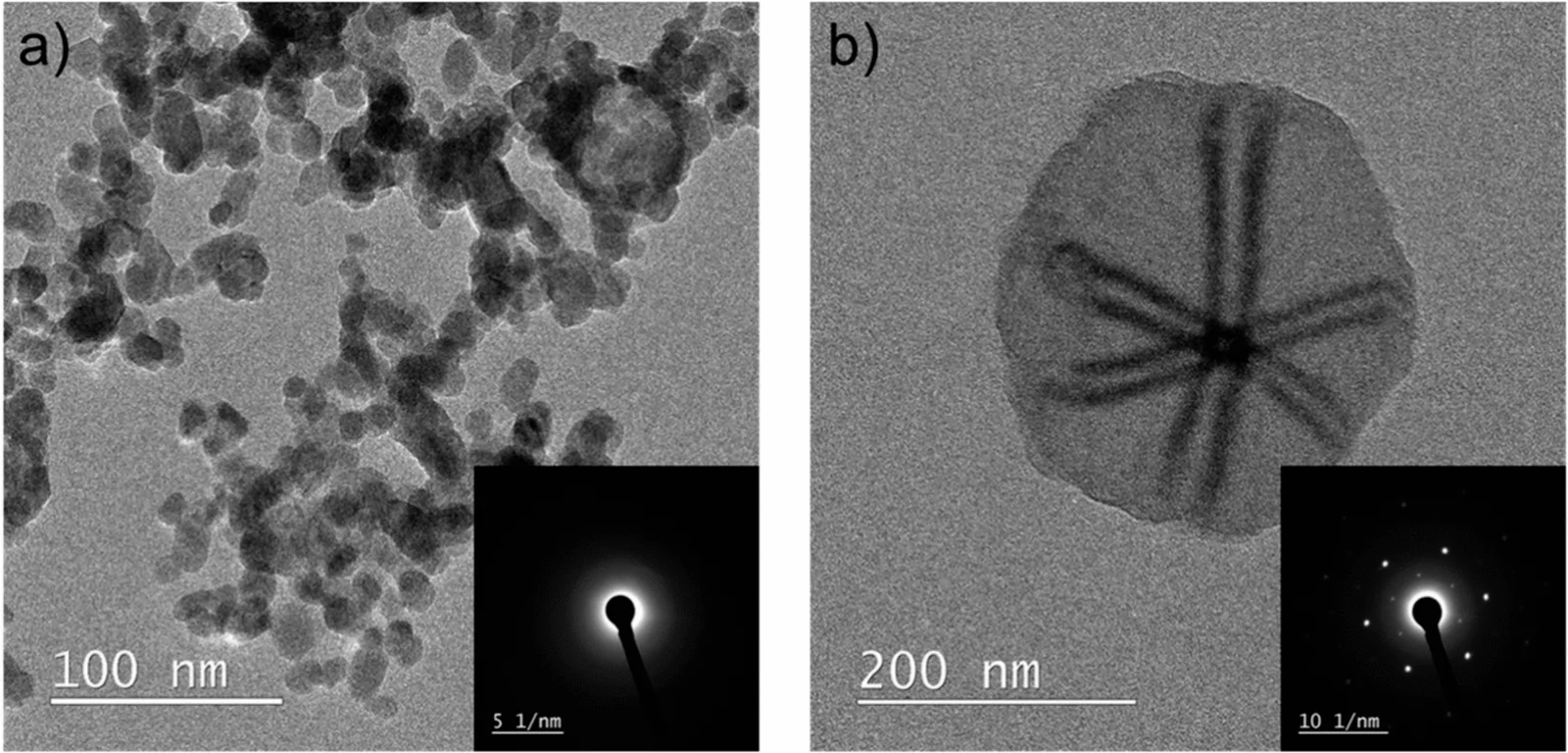
Characterization of particles formed during potentiometric titration without additives **a**–**b** employing transmission electron microscopy in a dry state at ambient temperature. The inserted electron diffraction patterns identify the particles as amorphous (**a**) or crystalline (**b**), with the observed reflections matching those expected for brucite in the latter case. Note that the cross-like variations in electron contrast observed for the crystal in **b** may arise due to local differences in thickness (for a hexagonal-bipyramidal habit) and/or bending contours caused by tensions in the basal plane. Reproduced with permission from [90]

As MgO dissolves, the solution reaches supersaturation, and the precipitation of Mg(OH)_2_ can occur. *Classical crystallization* theory treats this process as a direct transition from dissolved ions (Mg^2+^ and OH^−^) to the crystalline Mg(OH)_2_, in which ions directly form ordered crystal structures [88]. However, recent investigations across diverse mineral systems reveal that precipitation rarely follows a direct path from dissolved ions to solid crystals. Instead, it typically proceeds through intermediate stages: initial clustering of ions, formation of liquid-like droplets, solid disorder (amorphous) precursors, and gradual assembly of particles into solid structures. These observations have laid the foundation for a novel view of crystallization, termed *non-classical*, encompassing mechanisms that do not proceed exclusively through atomic/molecular building units [89], (Fig. 6).

In the Mg(OH)_2_ system, this *non-classical framework* is also relevant. Rather than the simplistic nucleation-and-growth model proceeding solely via ion-ion attachment to crystalline nuclei, Mg(OH)_2_ precipitation involves several key steps [91]. The process begins with a pre-nucleation stage featuring free ions, ion pairs, complexes, and larger clusters. Analysis of the ratio of bound Mg^2+^ to OH^−^ ions suggests that the pre-nucleation associates may carry a net positive charge (MgOH^+^). The first solid phase that forms is a transient amorphous Mg(OH)_2_ phase (Fig. 6a), observed here and in two studies using completely different experimental conditions [92, 93]. Brucite then crystallizes from this amorphous precursor (Fig. 6b) [91].

Understanding brucite precipitation as a multistep pathway opens possibilities for targeted control [94]. Each stage, from free ions and ion clustering to amorphous precursors and the crystalline phase, may offer intervention points to prevent MgO surface passivation by manipulating Mg(OH)_2_ precipitation.

### Towards controlling Mg(OH)_2_ precipitation

#### Thermodynamic control through Mg-complexation

Mg complexation can shift brucite precipitation from the MgO surface to the bulk solution. By forming complexes with Mg^2+^, additives reduce free Mg^2+^ activity, lowering supersaturation with respect to brucite at the interface and impeding surface nucleation. This also increases undersaturation with respect to MgO, promoting dissolution and enabling Mg^2+^ diffusion into the bulk for precipitation away from the surface. Different anions vary in their complexation strength with Mg^2+^, and understanding these differences is essential for selecting effective additives.

To classify the impact of the different anions, the thermodynamic modelling of brucite solubility in the presence of different anions was carried out using GEM-Selektor (GEMS) [95, 96]. General thermodynamic data were taken from the PSI Nagra database [72], data for the complex formation of magnesium ions with different anions were added as detailed in Table 1. The activity coefficients of the aqueous species were calculated with the built-in extended Debye-Hückel equation:

9 $$\mathrm{log}{\gamma }_{i}= \frac{{-A}_{y}{z}_{i}^{2}}{1+ {B}_{y}a\sqrt{I}}+ bI$$

and NaCl as a background electrolyte with a common ion size parameter *a* = 3.31 and *b* = 0.98 [96, 97]. z_i_ represents the charge of species *i*, I the effective ionic strength and *A*_*y*_ and *B*_*y*_ the *P,T*-dependent Debye-Hückel solvent parameters. The titrations were carried out with HCl and NaOH, except for complexation with Cl^−^, for which HNO_3_ was used instead. Counterions were present, increasing the ionic strength in a similar manner across all calculations, except for the pure water.

**Fig. 7:**
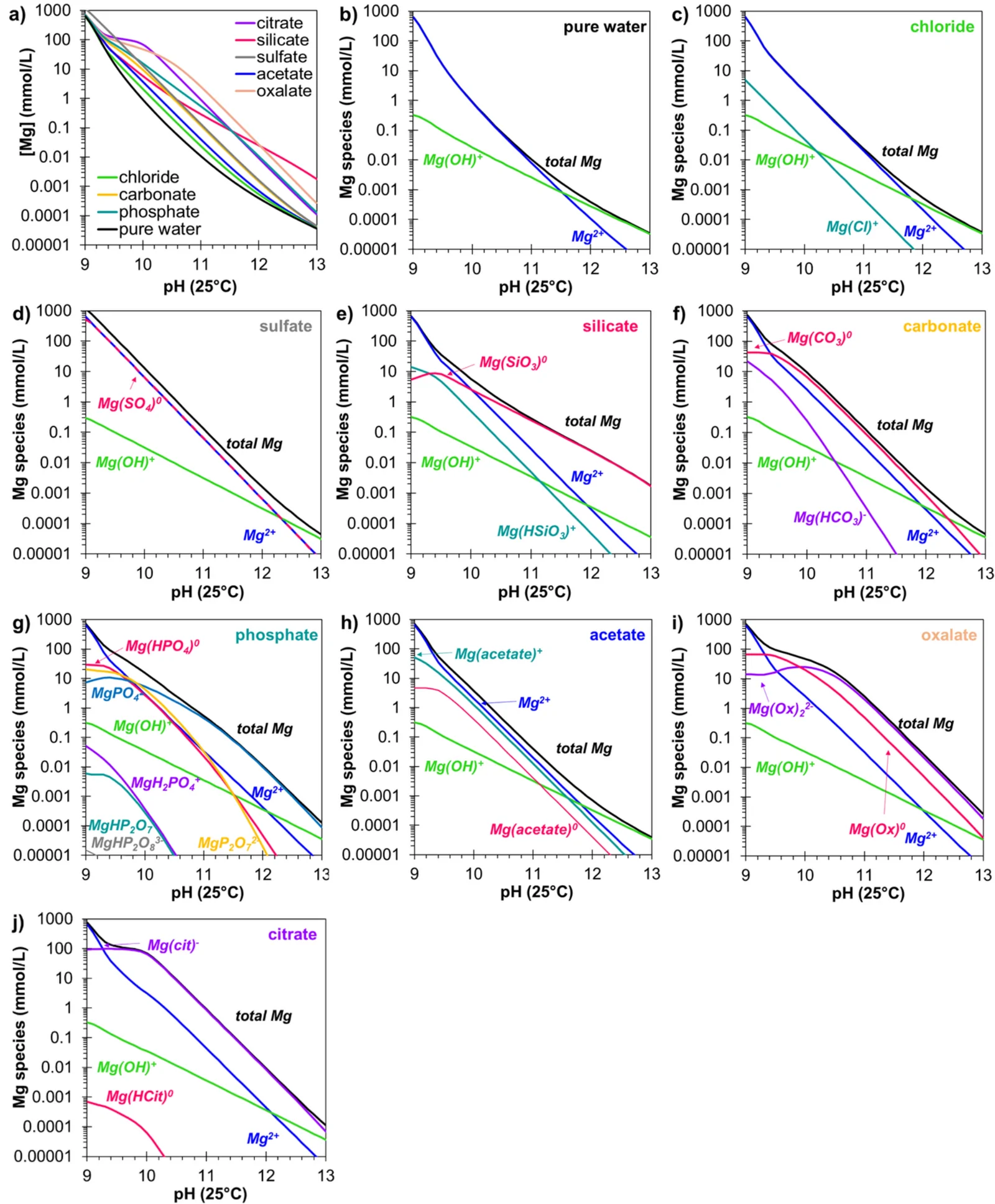
**a** Modeled brucite solubility curves for the different additives (20 g of brucite in 100 g of water, additive = 100 mmol. All solids, except brucite, are prohibited from forming. Details on the speciation of the Mg are given for **b** pure water, **c** chloride solution, **d** sulfate solution, **e** silicate solution, **f** carbonate solution, **g** phosphate solution, **h** acetate solution, **i** oxalate solution, **j** citrate solution

The solubilities of Mg(OH)_2_ with set anion concentrations of 100 mmol/L are presented in Fig. 7a. In pure water (Fig. 7b), the solubility is strongly influenced by the pH of the environment. Between pH 9–13, the total concentration of Mg drops by 7 orders of magnitude. This indicates the low solubility of Mg(OH)_2_ in high alkalinity in contrast to that of Ca(OH)_2,_ which is 6 orders of magnitude higher in total Ca concentration at pH 13 (see supplementary information, Figure S1). In pure water, there are 2 aqueous species of Mg (i.e., Mg^2+^ and Mg(OH)^+^). Among the two species, Mg^2+^ dominates until pH ca. 11.5, whereas Mg(OH)^+^ is the main species at high pH. Chloride does not clearly alter the solubility of Mg(OH)_2_ (Fig. 7c) and this is due to the very weak complex of MgCl^+^ as shown in Fig. 7b. However, the dissolved Mg is slightly higher in the presence of NaCl compared to the pure water due to the increase in the ionic strength of the solution. The concentration of Mg can theoretically be increased by the presence of dissolved silica as two complexes concerning Mg and dissolved silica are reported in literature: MgHSiO_3_^+^, MgSiO_3_^0^. The modeling of the Mg solubility of brucite (Fig. 7d) in the presence of dissolved silica showed that if only brucite is the solid precipitated, the Mg concentration can be increased. However, the complexes are forming at a rather high pH (above 9.5) and the M–S–H precipitation, experimentally observed, reduces strongly both the dissolved Mg and Si [98].

The bicarbonates and carbonates complexes with the dissolved magnesium ions are reported in the PSI Nagra database [72], and similar complex formation constants were reported in [57]. Figure 7a shows an increase of the dissolved Mg ions in the presence of carbonates. Similarly, the Mg concentrations were experimentally observed to also increase in the presence of carbonates for suspension at equilibrium with M–S–H samples [99]. The formation of Mg-carbonate complexes is stronger compared to the Mg-bicarbonate complexes, and in the range of interest, the dissolved carbon is mostly present as carbonates (Fig. 7f). Phosphates are also reported to increase the Mg in solution [100, 101]. The strong complexes between the different phosphate species and magnesium ions reported [74] results in a much higher dissolved Mg at equilibrium with brucite. The most important complexes are the MgPO_4_^−^ and MgP_2_O_7_^2−^ (Fig. 7g) [74].

The use of organic additives can also increase brucite solubility to different degrees (Fig. 7h–j). Here, we assessed three organic ligands, namely acetate, citrate, and oxalate. Among the three, citrate and oxalate drastically change the solubility of brucite via the strong complexes of Mg-citrate and Mg-oxalate. In the presence of citrate, Mg(cit)^−^ is the most dominating complex in a wide range of pH (ca. 9.4–13), leading to an increase in Mg concentration by ca. 2 orders of magnitude compared to pure water. Similarly, oxalate forms two strong complexes with Mg, i.e., Mg(Ox)_2_^2−^ and Mg(Ox)^0^, which are dominating species in a similar pH range to that in the Mg-acetate system. Between citrate and oxalate, the former seems to have stronger effects on Mg(OH)_2_ solubility at pH < 10.5, while the latter works at a higher pH range (pH > 11). In contrast, acetate offers only a modest increase in the solubility of brucite. This is attributed to the relatively weak complex of Mg(acetate)^+^ compared to the other complexes of Mg with citrate or oxalate. Notably, the Mg(acetate)^+^ complex is also weaker than the activity of Mg^2+^ in this system. However, the open literature recorded the use of acetate as an important additive to enhance the performance of magnesium carbonate cement. Acetate is known to enhance the hydration kinetics of MgO and brucite precipitation [102] or to improve carbonation of brucite and mechanical performance [46, 81, 103]. Therefore, it will be advantageous to further investigate the use of citrate and oxalate as additives in a similar manner as acetate and understand the role of these ligands in MgO-based cements.

**Fig. 8:**
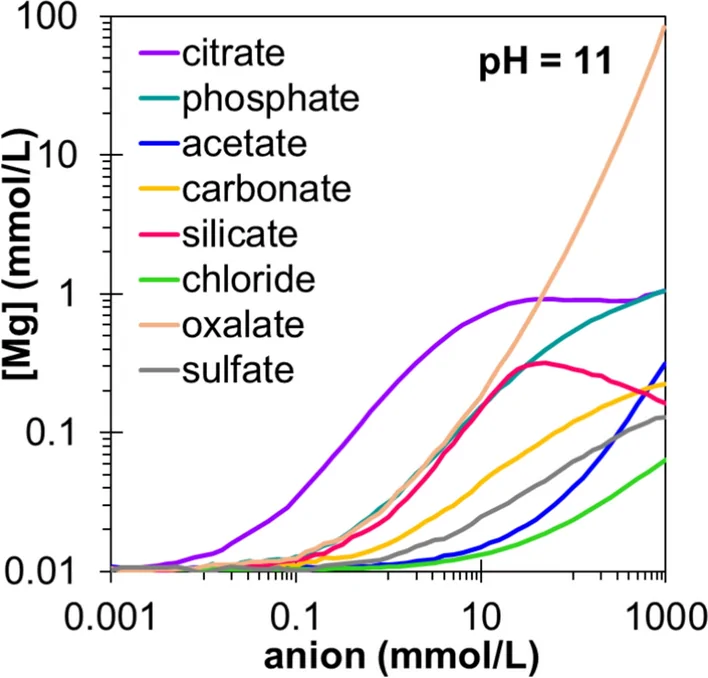
Magnesium concentration versus anion concentration at a set pH of 11. Note that solid precipitations were prohibited except for brucite precipitation. However, after a certain concentration, the precipitation of solids from Mg and the specific anions would occur, such as M–S–H above [Si] = 2 mmol/L

In Fig. 8, we further assessed the total Mg concentration in relation to different concentrations of anions added to the fixed pH 11 solution. Since pH 11 is a common pH value in many magnesia-based cementitious systems, the relationship between Mg concentration and the dosage of additives added to the system may provide insight into suitable selection and dosages of these additives. From all organic and inorganic anions (of this study), citrate exhibits the most noticeable influence on enhancing the Mg concentration in solution, even at small citrate content. In addition, oxalates, phosphates, and silicates have similar contributions to the increase of Mg content in the solution when the anion concentration is below 10 mmol/L. Above this concentration, oxalate exhibits a remarkable increase in Mg content, whereas phosphates and silicates appear to have either a slight increase or decrease in Mg content, respectively, compared to their effects at lower concentrations. Notably, solids of Mg with these anions might form at these high concentrations, whose precipitation we excluded in the present calculations. In contrast, the addition of chloride shows a negligible increase in Mg concentration in the solution, while acetate and carbonate may enhance the Mg concentration at relatively high dosages of these anions. It is to note that while salts like Mg(acetate)_2_, Mg(formate), and others present high solubilities, the precipitation of M–S–H, Mg-hydroxy-carbonates or Mg-phosphate phases as newberyite and bobierrite might precipitate long before reaching such high silicate, carbonate or phosphate concentrations presented in Fig. 7. Hence, the precipitation of M–S–H and other cementing phases in the presence of additives requires further study and constitutes the focus of the next section.

#### Controlling precipitation location

As chemical additives are used to shift brucite precipitation from the MgO surface to the bulk solution, their appropriate selection is crucial, as they should not strongly adsorb to the MgO surface and impede its dissolution. Chemical additives and pH together control where Mg(OH)_2_ nucleates and precipitates, preventing passivation at the MgO particle surface. Hence, different additives are posited to have distinct mechanisms to shift nucleation from the surface toward the bulk solution.

Carboxylic-based additives form stable complexes with Mg^2+^, such as ethylenediaminetetraacetic acid (EDTA) and citric acid, and significantly influence MgO dissolution through distinct mechanisms. In dead-burned magnesia, tartaric and citric acid preferably adsorb on the MgO surface; while EDTA exhibits high affinity to Mg^2+^ ions instead of adsorbing on the MgO surface [104]. EDTA binds alkaline-earth ions in a hexacoordinated manner, leading to complexes with *C*_*2*_ symmetry and approximately octahedral coordination [105]. While the complexation does not directly inhibit Mg(OH)₂ precipitation, as might be expected due to the stability of Mg^2^⁺-EDTA complexes, it alters the ultimate precipitation location, favoring homogeneous nucleation in solution. Acetate has been suggested to operate through a similar mechanism, forming magnesium-acetate complexes that migrate away from the MgO surface and dissociate in bulk solution, thereby promoting Mg(OH)_2_ bulk precipitation rather than surface nucleation [79]. In contrast, citric acid exhibits a higher affinity for adsorption onto the MgO surface, thereby hindering MgO dissolution and consequently reducing Mg(OH)₂ precipitation [104]. This surface-blocking effect is not ideal in MBCs, as it limits the release of Mg^2+^ building units required for the precipitation of the binding phase. However, careful control of citric acid dosage may allow exploitation of its strong complexation properties while reducing surface adsorption effects.

#### Precipitate morphology control

Morphology control is critical because Mg(OH)_2_ particle shape can influence both dissolution behavior and passivation of the MgO surface. Flaky or platelet morphologies provide greater surface area, potentially enhancing dissolution when Mg(OH)_2_ serves as a Mg source. Equally important, morphology determines how particles pack at the MgO surface, i.e., open structures maintain porosity for continued Mg-ion transport, while dense arrangements create impermeable barriers that halt dissolution.

The pH during MgO hydration plays a critical role in regulating the morphology of Mg(OH)₂ particles. A notable change in the morphology of brucite crystals occurs at a pH of about 11.5. Below this threshold, the crystals typically form thin, sheet-like structures with a flaky appearance. However, when the pH rises above 11.5, the growth pattern changes significantly, resulting in more robust columnar crystals with a blockier shape [67].

**Fig. 9:**
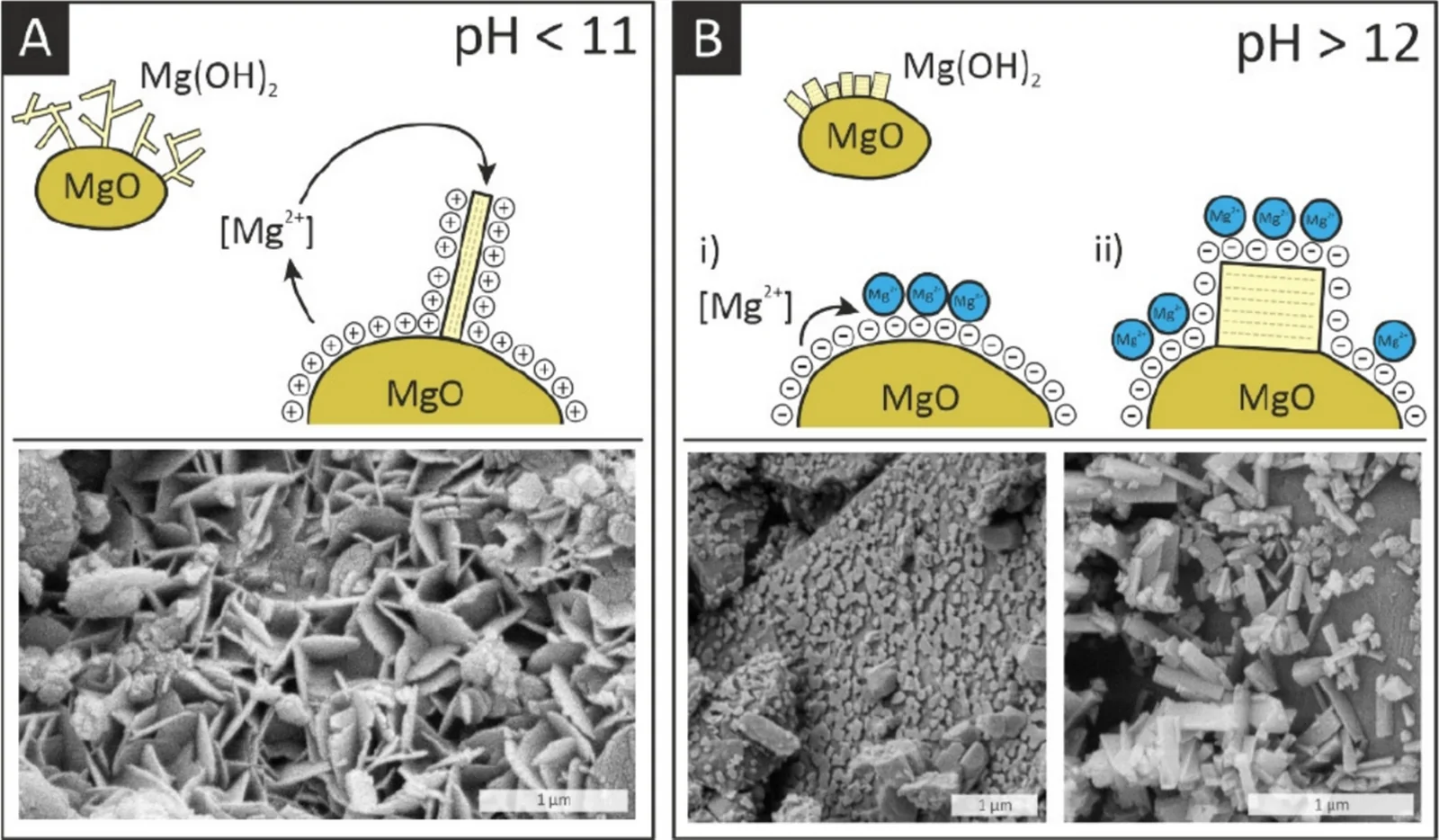
Conceptual model and representative SEM images of the hydration products formed at pH levels **A** below, and **B** above the pH_PZC_ of MgO (10.8–12). Reproduced with permission from [67]

The authors propose that brucite morphology is primarily influenced by two pH-dependent factors: (1) variations in the surface charge of reactive MgO across different pH levels; and (2) the predominance of distinct nucleation mechanisms. In this context, the point of zero charge (PZC) of MgO has been experimentally found to lie in the pH range of 10.8–12.0 [106] and calculated from DFT simulations at a pH of 11.44 [107]. Above the PZC, negatively charged species from deprotonated ≡Mg–O dominate the MgO surface. These attract Mg^2+^ ions, leading to localized and heterogeneous nucleation and growth of columnar, block-shaped Mg(OH)_2_ (Fig. 9B). While below the PZC, positively charged surfaces (e.g., ≡MgOH^+^) repel Mg^2+^ ions, promoting the homogeneous nucleation of Mg(OH)_2_ particles, which, upon precipitation and crystallization on the MgO surface, grow to their flaky appearance (Fig. 9A).

Anionic additives also substantially alter the morphology of the resultant Mg(OH)_2_. For instance, the formation of Mg-acetate complexes, as already discussed, affects not only the location of precipitation but also the morphology, producing uniform hexagonal Mg(OH)_2_ plates that agglomerate into blossom-petal-like aggregates [79, 108]. The hydration of MgO in the presence of chloride ions, with a much lower affinity for Mg (Fig. 7c), results in elongated hexagonal prisms [108].

The presence of sodium hexametaphosphate during the hydration of MgO was recently observed to produce irregular, blocky Mg(OH)_2_ crystals due to rapid precipitation and surface adsorption, whereas sodium carbonate limited c-axis growth of brucite, generating thin flakes of Mg(OH)_2_ [109].

Higher-molecular-weight additives, such as polyacrylic acid, a common anti-scalant, operate through a different mechanism that does not appear to rely primarily on Mg^2^⁺ complexation [91]. Polyacrylic acid significantly delays brucite nucleation and alters particle morphology, leading to smaller nanoplatelets. After nucleation, the polymer exhibits strong interactions with the solid phase and inhibits growth by crosslinking particles, yielding brucite nanoplatelets intimately interspersed with coprecipitated organic polymer[91]. The growth inhibition of brucite, along with the resulting high surface area and reduced particle size, is promising for enhancing subsequent brucite dissolution and forming binding phases in MBCs.

#### Precipitate crystallinity and phase modification control

The crystallinity of Mg(OH)_2_ directly influences its solubility and reactivity in MgO-based cements. Lower-crystallinity phases exhibit higher solubility, thereby enhancing dissolution and Mg^2+^ availability for binding phase precipitation.

##### Low-crystalline and amorphous phases

Amorphous and low-crystalline phases often form preferentially over their crystalline counterparts due to kinetic factors [110]. Although crystalline Mg(OH)₂ is thermodynamically more stable, amorphous phases, characterized by non-stoichiometry, strong hydration, and instability, can nucleate more readily because they have lower interfacial energy barriers [111]. Under conditions of high supersaturation or in the presence of nucleation-inhibiting additives, the system favors the rapid precipitation of these metastable phases [112], which subsequently transform into more stable crystalline structures. Such amorphous Mg(OH)₂ precursors have been identified in multiple contexts: during the in-situ hydration of MgO as observed by liquid cell transmission electron microscopy (TEM) [92], on MgO surfaces exposed to humid air [93], and during homogeneous precipitation of Mg(OH)₂ at room temperature [91]. These observations demonstrate that amorphous intermediates play a role in brucite precipitation whether it nucleates at MgO surfaces or in bulk solution.

Additives may influence crystallinity through several mechanisms. Direct incorporation into the precipitating phase represents one pathway, as observed with magnesium acetate, which promotes the precipitation of Mg(OH)_2_ as low-crystalline, sheet-like layers with nanometer-scale thickness and large diameters [113]. Inorganic species such as carbonate can also modify Mg(OH)_2_ crystallinity through incorporation into the crystal structure [114, 115], as discussed below. Additional mechanisms observed in other mineral systems include nucleation inhibition, where additives increase local concentration of ions and favor amorphous phase precipitation [116], and water stabilization, where additives hinder dehydration and prolong amorphous phase lifetime [117]. These mechanisms may operate simultaneously or independently depending on conditions, though their relevance to Mg(OH)₂ systems remains to be systematically investigated. Moreover, fundamental questions persist regarding the composition and structure of amorphous Mg(OH)_2_, including the extent of water incorporation and its role in phase stability.

Despite these uncertainties, low-crystalline Mg(OH)₂ has enhanced carbonate product characteristics and carbon mineralization efficiency [113], demonstrating that crystallinity control can improve magnesium carbonate cement performance. This proof-of-concept supports systematic investigation of additive effects on Mg(OH)_2_ crystallinity and its influence on binding phase precipitation kinetics as a promising pathway for advancing MBCs.

##### Modified Mg(OH)_2_ crystallites through additive incorporation

Carbonate incorporation into Mg(OH)_2_ has received the most attention due to its central role in magnesium carbonate cements. When hydrated magnesium carbonates such as hydromagnesite [Mg_5_(CO_3_)_4_(OH)_2_ 4H_2_O] [118, 119] or nesquehonite (MgCO_3_·3H_2_O) [120] are present during MgO hydration, water molecules and/or carbonate ions intercalate between brucite layers, creating stacking fault of brucite [115], 114 that manifest as the splitting or broadening of the 001 reflection in XRD diffraction [115]. This modified structure is termed hydrous carbonate-containing brucite (HCB) [114].

The incorporation of water and carbonate is also influenced by temperature, as elevated temperatures (e.g., 60 °C) only enable the incorporation of carbonate in brucite’s structure while excluding water [115]. The precipitation of HCB was found to be more favorable than neat brucite in these blends, as the presence of hydrated magnesium carbonate phases provides the source of carbonate species in the systems where the HCB is known to be the strength-giving phase in magnesium carbonate cements [114, 120, 121]. In addition, this stacking fault in brucite can also be created by the addition of organic additives such as acetate [102], where distortions in the 001 reflection of the brucite were observed, similar to the case for HCB. Plus, the presence of acetate increases hydration kinetics and the degree of MgO, with significant changes in the phase morphology, as mentioned above.

Collectively, additive-mediated control over precipitation location, morphology, and crystal structure provides a framework for engineering Mg(OH)_2_ precipitation and transforming MgO surface passivation from a performance obstacle into a design tool for MBCs.

## Precipitation of magnesium silicate hydrates

The M–S–H in dilute systems, such as suspensions and slurries, has been studied in the last 30 years, particularly in the context of its kinetics of precipitation and stability [98, 122–128]. When reactive MgO is combined with amorphous SiO_2_, it initially forms Mg(OH)₂, creating a slightly alkaline environment with pH 11–11.2 [98]. Mg(OH)₂ dissolution gradually provides dissolved Mg^2+^ and OH^−^ ions, which then react with the silicate species from amorphous SiO_2_. This sequential process governs the precipitation of magnesium silicate hydrates in MgO-SiO_2_ systems. Although Mg(OH)₂ is thermodynamically less stable than M–S–H, its prior precipitation hinders M–S–H precipitation due to its slow dissolution and/or low solubility. M–S–H precipitation is notably slower than calcium silicate hydrate (C–S–H) precipitation from the reaction of CaO and SiO_2_ in suspension. While this was attributed to the dilute systems, pastes containing only reactive MgO and SiO_2_ also showed slow precipitation kinetics [129]. The slower rate can be explained by the lower solubility of Mg(OH)₂ at pH levels above 10.5, with very low dissolved Mg at equilibrium. In contrast, as explained earlier, calcium hydroxide (Ca(OH)₂) has a higher solubility even at high pH levels of 12.5, reacting more readily with silicate species. Therefore, to accelerate M–S–H precipitation, strategies should be developed to enhance the concentrations of dissolved Mg and Si in the pore solution.

### Effect of chemical additives on M–S–H precipitation

Similarly to the MgO hydration, various chemical additives have been investigated to enhance the precipitation kinetics of M–S–H, with dissolved silicate species showing significant promise. In one study, adding a relatively small amount (2.5 wt.%) of sodium metasilicate to MgO-SiO_2_ slurries significantly suppressed Mg(OH)₂ precipitation compared to a reference mix, thereby promoting M–S–H at early ages [130]. This benefit can be attributed to Mg-Si complexes that increase Mg availability for M–S–H precipitation at the high-pH environment, which otherwise would lower Mg availability. At longer curing times, sodium metasilicate further accelerates the conversion of Mg(OH)₂ to M–S–H [130, 131]. These findings suggest that other chemical additives offering comparable complex-forming capabilities at elevated pH may similarly enhance Mg availability, ultimately improving M–S–H precipitation in MBCs.

Phosphates are also known to increase Mg concentration significantly due to their strong affinity to Mg^2+^. A specific phosphate salt, sodium hexametaphosphate (NaHMP), often used as a superplasticizer in magnesia-silicate-based cements [132], showed that in addition to increasing Mg availability, the phosphates adsorb on the MgO surface and inhibit the heterogeneous nucleation of Mg(OH)₂ [133]. In addition, the pH is increased and enhances the dissolution rate of SiO₂ to form M–S–H [134]. Tonelli et al. demonstrated that polyphosphate additives undergo hydrolysis at the pH of the MgO-SiO_2_ system, resulting in ortho- and pyrophosphates as the main active species. The study established a clear correlation between the increase in M–S–H precipitation and the concentration of orthophosphate species, suggesting that this may be an effective additive to be used in M–S–H [135]. Further studies have reinforced orthophosphate's effectiveness, concluding that the high pH it induces is the key factor driving enhanced M–S–H precipitation [136]. Using sodium carbonate salts [137] and reactive Mg-carbonate salts [138] also improved the precipitation of M–S–H. This has been primarily attributed to the complexation of Mg^2+^ by carbonate species, the destabilization of brucite in the case of the highly soluble sodium salt, and the provision of additional nucleation sites for M–S–H by Mg-carbonate seeds [138]. Additionally, the addition of sodium carbonate to MgO also increases the pH of the suspensions [134], hence, it can promote the precipitation of the M–S–H.

### Effect of chemical additives on the nucleation and growth of M–S–H

These results already highlight the promoting effect of some additives on the nucleation and growth of M–S–H; however, research has not yet fully established if these enhancements are limited to influencing the hydration reaction of MgO or the nucleation of M–S–H itself. In the absence of chemical additives, prior to the homogeneous nucleation of M–S–H, a complex mixture of hydrated magnesium-(sodium)-silicate oligomers exists in solution [139], resembling the nucleation pathway seen in C–S–H cement systems [140]. These oligomers aggregate, forming an ill-defined, liquid-like M–S–H precursor phase that gradually transforms into a denser M–S–H network characterized by nano-globular particles with a distinct sheet-like surface structure [139]. When chemical additives are used in magnesium silicate cement to raise the magnesium concentration in the solution, it is essential that these additives do not significantly hinder M–S–H nucleation. Ideally, while they form complexes with Mg species, they should avoid sequestering it to a level that makes it unavailable for M–S–H growth.

**Fig. 10:**
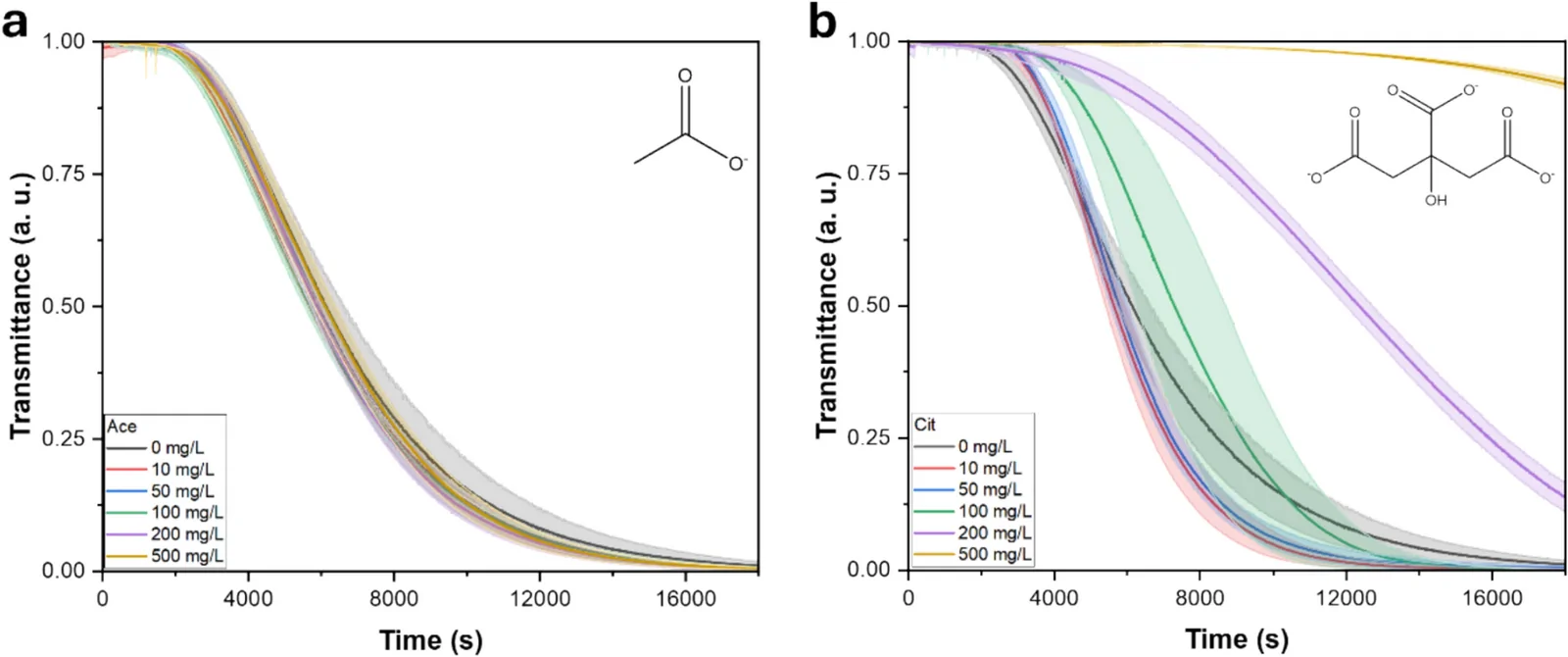
Time-dependent evolution of solution transmittance during M–S–H nucleation and growth in the presence of **a** acetate (Ace) and **b** citrate (Cit) at different concentrations. Initially, the clear solution exhibits maximum transmittance, which decreases upon M–S–H nucleation takes place. While increasing acetate concentrations show negligible effect on transmittance evolution, citrate significantly slows the decrease. Reproduced with permission from [141]

Depending on their ability to form stable Mg-complexes, chemical additives significantly influence the M–S–H nucleation and growth process. In recent investigations [141], the effects of four anionic additives (acetate, citrate, orthophosphate, and carbonate) on the homogeneous nucleation of M–S–H were explored using a standardized crystallization protocol. Among the tested anions, acetate showed minimal impact on M–S–H nucleation and growth across all concentrations (Fig. 10a), neither delaying nucleation nor altering the characteristics of the resulting M–S–H material. This aligns with acetate’s tendency to form relatively unstable complexes with Mg. In contrast, citrate was the most influential additive (Fig. 10b), delaying nucleation even at low concentrations and significantly altering the growth by stabilizing an M–S–H precursor phase, especially at higher concentrations. Orthophosphate, at low concentrations, appeared to enhance M–S–H nucleation. However, a magnesium phosphate phase initially precipitated beyond a certain phosphate threshold but then transformed into M–S–H over time. Sodium carbonate, meanwhile, consistently provided a mild enhancement of M–S–H nucleation and growth across all tested concentrations, likely due to the increased pH of the initial solutions. Importantly, none of the tested chemical additives caused significant delays in M–S–H nucleation, a favorable outcome for practical applications. Moderate concentrations of phosphate and carbonate are especially promising for practical use, as they seem to reduce nucleation barriers with minimal impact on the overall precipitation process.

The intricate interplay between chemical additives and M–S–H nucleation and growth is multifaceted, as evidenced by non-linear relationships between additive concentrations, Mg(OH)_2_ solubility (Figs. 7 and 8), and subsequent M–S–H precipitation pathways (Fig. 10). Although certain complexation agents can significantly enhance the solubility of Mg(OH)_2_, this benefit does not always translate to enhanced M–S–H nucleation. For example, at low doses, citrate (< 10 mmol) can increase Mg(OH)_2_ solubility, but also modifies MgO dissolution rate through surface adsorption, while also influencing Mg(OH)₂ nucleation [104]. In other cases, pre-nucleation interactions between chemical additives and dissolved Mg or silicate ions may encourage M–S–H precipitation kinetics and even form Mg-complexes that act as nucleation centers. While this review demonstrates that complexation agents can alter these processes, many open questions remain about optimizing additive type and dosage, especially as the MgO crystal structure varies with production routes [142]. Further research into the mechanistic details of how these molecules influence the nucleation and growth of Mg(OH)₂ at the MgO particle, as well as their interactions with Mg species, silicates, and growing M–S–H phases in the bulk solution, will be crucial for tuning M–S–H cement formulations and advancing MBCs more broadly.

## Conclusions

MgO-based cements (MBCs) are a promising class of alternative cements due to their beneficial material properties and potential for decarbonization. While the production of MgO leads to important physicochemical properties that affect its hydration, significant research has been conducted to date to understand the hydration, solubility, and precipitation mechanisms in MBCs. In particular, the effects of chemical additives and aqueous chemical conditions (e.g., pH) are important areas of study to understand how MgO hydration and Mg(OH)_2_ solubility influence their nucleation and growth pathways, leading to M–S–H precipitation, either heterogeneously at the particle surface or homogeneously in the bulk solution.

Significant differences arise between MBCs and traditional calcium-based cement systems. While MgO and CaO share similar crystallographic structures, their hydration kinetics are vastly different, largely due to the stronger Mg-O bonds of magnesia and low solubility of Mg(OH)_2_, which forms a diffusive barrier on the MgO particle surface. To better understand this behavior, the extent of MgO surface hydroxylation is a key step governing the MgO-water interface during hydration. Recent studies indicate that a reconstructed MgO surface model with distinct solvated Mg^2+^ ions likely represents the MgO surface at the early stages of hydration. Dissolution is influenced by the developing surface potential across the particle surface and the bulk solution, as well as transport by concentration gradients of solvated species in solution, both of which can be affected by the use of chemical additives. Future work is needed to extend the hydration kinetic model of MgO into cement conditions. This work must be extended to also include the role of chemical additives capable of complexing with dissolved Mg species; reducing the rapid precipitation of low-solubility phases at the dissolving particle surface; and, increasing their effective Mg concentration. Chemical additives, therefore, can effectively increase the diffusive flux of dissolved Mg species.

As solvated Mg^2+^ ions diffuse to the bulk solution, the precipitation of Mg(OH)₂ involves non-classical crystallization pathways, with nucleation and growth occurring both homogeneously within the bulk solution and heterogeneously on the surface of the MgO particle. In this context, the effect of chemical additives is important as these can strongly adsorb onto the MgO particle, hence affecting Mg(OH)₂ nucleation and, subsequently, M–S–H growth. Here, insights from other disciplines, such as biochemistry and geochemistry, into the selective binding and complexing agents (e.g., proteins, microbes, and organic ligands) with Mg^2+^ offer great potential to develop suitable additives for MgO-based cements. Future work should further validate the chemical additive adsorption and influence on M–S–H precipitation in cement-relevant conditions with time-resolved characterization techniques. Another important factor in the nucleation and growth of Mg(OH)₂ is pH, which requires further research in cement-relevant conditions, as it has been shown to affect brucite morphology depending on the side of the point of zero charge (pH < 10.8 or pH > 12). Thus, pH may significantly influence the homogeneous nucleation process, as evidenced by the presence of amorphous precursors and intermediates before Mg(OH)₂ crystallization. Engineering this nucleation pathway may offer unique opportunities to further modify the precipitation of Mg(OH)₂ and M–S–H in MBC.

Thermodynamic simulations of various complexation agents demonstrate that across pH (9–13), carbonates and citrate increase the solubility of Mg(OH)₂ significantly, with silicon species being most effective at pH > 12. These results align with experimental findings where dissolved silicon species enhance the M–S–H precipitation kinetics. In a similar case, phosphates and carbonates are also shown to influence pH and increase Mg availability. However, their adsorption onto the MgO surface and complexing effects to increase the mobility of solvated Mg ion species remain important aspects for further study. While phosphates adsorb to the Mg(OH)_2_ surface and inhibit Mg(OH)₂ heterogeneous nucleation, citrates strongly adsorb onto MgO, hindering MgO dissolution. Overall, the interplay between particle-level hydration and bulk-solution chemistry remains underexplored. This state-of-the-art review underscores the significant research opportunities in fine-tuning MBC hydration, transport, and precipitation pathways. By addressing these open questions, researchers can advance MBC formulations toward widespread use, furthering the push for low-carbon alternatives in cement and concrete technology.

## Supplementary Information

Below is the link to the electronic supplementary material.

Supplementary file1 (DOCX 4599 KB)

## Data Availability

This is a review manuscript; hence, the data are given in Table 1 or are available elsewhere.
