# Supplementary material for: Dissolution, diffusion, and precipitation of Mg2+ in MgO-based cements, a review by the RILEM TC-311 MBC
Source: Mater Struct. 2026 Mar 23;59(3):161. doi: 10.1617/s11527-026-03048-x (PMC13668919; doi:10.1617/s11527-026-03048-x)
Supplement: Supplementary file 1 [file 11527_2026_3048_MOESM1_ESM.docx]

**Supplementary information**

**Figure S1:** Calcium speciation sas a function of pH at room temperature.
